# Stem Cells and Cancer Stem Cells: The Jekyll and Hyde Scenario and Their Implications in Stem Cell Therapy

**DOI:** 10.3390/biomedicines9091245

**Published:** 2021-09-17

**Authors:** Wan Safwani Wan Kamarul Zaman, Asma Abdullah Nurul, Fazlina Nordin

**Affiliations:** 1 Department of Biomedical Engineering, Faculty of Engineering, Universiti Malaya, Kuala Lumpur 50603, Malaysia; 2 Centre for Innovation in Medical Engineering (CIME), Department of Biomedical Engineering, Faculty of Engineering, Universiti Malaya, Kuala Lumpur 50603, Malaysia; 3 School of Health Science, Universiti Sains Malaysia, Kubang Kerian 16150, Kelantan, Malaysia; nurulasma@usm.my; 4 Centre for Tissue Engineering and Regenerative Medicine (CTERM), Universiti Kebangsaan Malaysia Medical Centre, UKM, Cheras, Kuala Lumpur 56000, Malaysia; nordinf@ppukm.ukm.edu.my

**Keywords:** stem cells, stem cell therapy, cancer stem cells, cancer cell, biosafety, bioefficacy

## Abstract

“Jekyll and Hyde” refers to persons with an unpredictably dual personality, who are battling between good and evil within themselves In this regard, even cells consist of good and evil counterparts. Normal stem cells (NSCs) and cancer stem cells (CSCs) are two types of cells that share some similar characteristics but have distinct functions that play a major role in physiological and pathophysiological development. In reality, NSCs such as the adult and embryonic stem cells, are the good cells and the ultimate treatment used in cell therapy. CSCs are the corrupted cells that are a subpopulation of cancer cells within the cancer microenvironment that grow into a massive tumour or malignancy that needs to be treated. Hence, understanding the connection between NSCs and CSCs is important not just in cancer development but also in their therapeutic implication, which is the focus of this review.

## 1. Introduction

Stem-cell therapy may provide an effective solution in regenerative and reconstructive medicine, particularly in the treatment of chronic diseases. One of the profound abilities of stem cells that makes them an ideal tool for cell-based therapy is the ability to differentiate into specific cell lineages depending on intrinsic or extrinsic stimulations. Promising benefits of stem cells have led researchers to embark on experiments and translating those outcomes for clinical application. With the advancement of biomedical technologies, innovative products derived from stem cells or to be used in stem-cell therapy can be utilised in treating patients, particularly with debilitating diseases. However, just as in any cell therapy, stem cells have complex characteristics [1] and their clinical application may pose challenges that require their biosafety to be addressed [2]. In regard to biosafety, the transformation of normal cells into cancer cells has always been a concern, particularly in stem-cell therapy, and it is normally discussed within the context of cancer stem cells (CSCs). CSCs are subpopulations of tumour cells with distinct stem-like properties that are responsible for tumour initiation, invasive growth, and metastasis formation [3,4].

**Figure 1:**
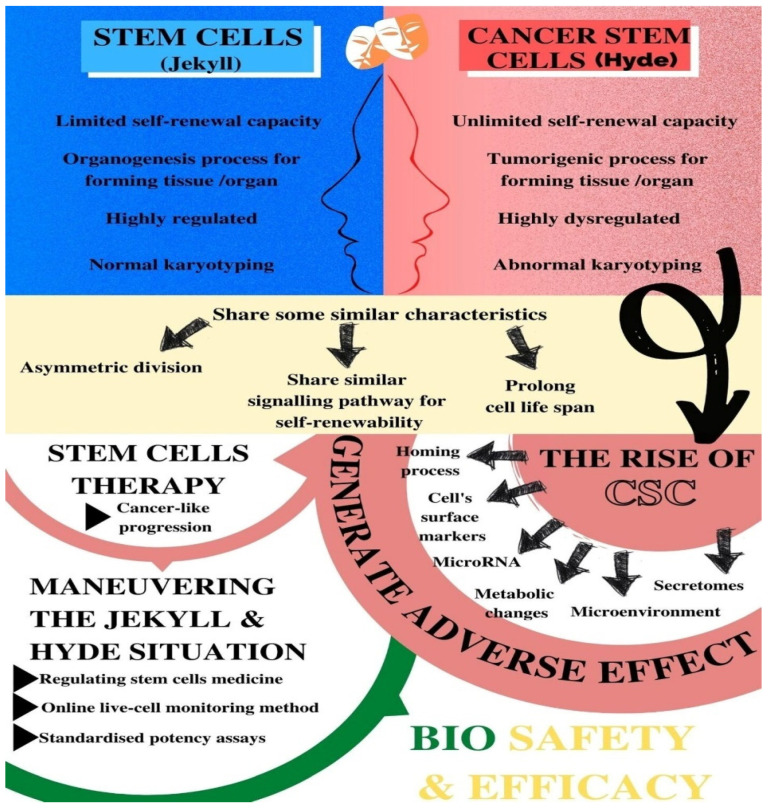
The overview of the “Jekyll and Hyde” scenario between stem cells and cancer stem cells and its implications on stem-cell therapy.

Stem cells or normal stem cells (NSCs) have been widely classified as embryonic stem cells (ESC), adult stem cells (ASC), and induced pluripotent stem cells (iPSC) that can share some similar characteristics with CSCs, such as the ability to self-renew and differentiate into other lineages [5,6]. In the event of cancer development, these characteristics of CSCs are the drivers of tumour growth and heterogeneity. Although quiescent, stem cells have the ability for self-renewal and differentiation into specific cell lineages, depending on appropriate biochemical and biophysical cues. There are three types of stem cells: embryonic stem cells, adult stem cells, and induced pluripotent stem cells (IPSC). Adult stem cells such as mesenchymal stem cells (MSCs) can be derived from different tissue sources such as adipose, bone marrow, and dental pulp [7]. They can be either unipotent or multipotent due to their tri-lineage differentiation ability whereby they can differentiate into chondrogenic, adipogenic, and osteogenic cells [7]. In comparison, IPSCs are somatic cells that are genetically reprogrammed to become embryonic-like stem cells by introducing genes that can maintain embryonic stem-cell characteristics [8]. Similar to cancer cells, stem cell characteristics also include heterogeneity and plasticity [9]. These two characteristics would also depend on the niche and microenvironment that the cells reside in that can very well promote stem-cell conversion into CSCs, which has led to investigations being carried out to find the connection in terms of mechanisms involved or markers that can be used to determine the occurrence of cell abnormalities [10]. Figure 1 illustrates the overview of stem cells and cancer stem cells and the implications of the “Jekyll and Hyde” [1] scenario towards the safety and efficacy of stem-cell therapy.

Hence, this will be the focus of this review, which is to canvass current developments on the connection between stem cells and CSCs in terms of conversion mechanisms, specific markers for characterisation, and factors influencing the formation of CSCs that can be used to detect the abnormalities. From here, we may be able to indicate the biosafety implications in stem cell therapy.

## 2. A Brief Insight into Stem Cells vs. Cancer Stem Cells (CSCs)

A very common debate about the origin of CSCs is still ongoing, whether cancer results from stem cells or it is determined by cells that have stem cell-like properties [8]. There are a few theories that have been postulated on the origins of CSCs. Amongst them, the relevant theories include: (i) transformation of stem cells existing in tissue, resulting in transformed growth and differentiation properties, (ii) transformation of a native pool of early precursors that reacquire self-renewal properties, (iii) sequence of effective mutations that render devoted transient-amplifying antecedent or differentiated somatic cells among a tissue immortal (de-differentiation), and (iv) amalgamation of circulating bone-marrow-derived stem cells with tissue-residing cells [11].

Nevertheless, both stem cells (SCs) and CSCs do have common characteristics. According to Bapat et al. (2010), both cells have a capacity for asymmetric cell division that produces a quiescent stem cell and a dedicated progenitor cell [12]. Their cell renewability is regulated by the similar signalling pathway of Wnt, Sonic hedgehog, MAPK, and Notch, including BMI-1 at the epigenetic level. They also have some long telomeres that increase activities that lead to the prolonged cellular life span. Both cells express similar surface receptors, which can indicate that stem-cell markers are associated with homing and metastases. Table 1 summarizes the common characteristics of normal SC and CSCs.

On the other hand, NSCs and CSCs also have distinguishing characteristics that are specific and unique to each. According to Cetin and Topcul (2012), one of their specific characteristics is self-renewal capacity, which in NSCs is extensive and limited in potential, and in CSCs is indefinite proliferation potential [13]. Furthermore, the tissue- or organ-forming capacity is different between these two cell types, whereby normal SC will develop through a process called organogenesis to form the internal organs of an organism at the end of the gastrulation process (three germ layers). In contrast, CSCs will undergo tumorigenesis through the hierarchy or stochastic model to form tumour tissues. The stochastic model explains that biologically, the tumour cells are the same and over time are genetically unstable and lead to the accumulation of genetically altered cells with more aggressive characteristics. This pool of altered tumours then further increases the tumour heterogeneity and progression [14]. The standard tumorigenesis of CSCs is more inclined towards a hierarchical model where only small and distinctive subpopulations of CSCs can initiate the cancer progression and growth [15]. Both stochastic and hierarchical models are reasonable systems that have been hypothesised to describe tumour heterogeneity and their relation to CSCs. The origin of CSCs and their mechanism, however, remains unclear. Normal stem cells or progenitor stem cells have been theorized as being the originator of cancer stem cells. This is based on the similarities of cell surface markers, phenotype, and function. The rise of CSCs has also been linked with normal somatic cells with altered genetic and heterotypic characteristics through epithelial–mesenchymal transition (EMT). This EMT causes stem-like characteristics as what has been found when EMT was induced in immortalized human mammary epithelial cells (HMLEs) [16]. In terms of differentiation capacity, normal SCs are highly regulated to form various types of functional cells or tissue, but that is highly dysregulated in CSCs. Normal SCs have normal karyotyping unlike CSCs, which have abnormal karyotyping with genetic alterations. Table 2 summarizes the characteristics differences between normal SCs and CSCs. The details of CSCs’ characteristics will be further discussed in the next sections of this review.

## 3. Characteristics of Cancer Stem Cells (CSCs)

Over the past few years, cancer treatments have evolved, aiming for safer, less aggressive and more precise treatments. However, limitations of current treatments may be due to heterogeneity that causes CSCs to be resilient towards chemotherapy. The combination of chemotherapeutic agents, surgery, and radiotherapy has been effective in treating many types of cancer, but because of their non-specific nature, these treatments can also induce more heterogeneity, not just in the cancer cells but to the surrounding normal cells, leading to chemoresistance and limiting the efficiency of chemotherapy [17]. This may lead to the formation of CSCs, and the conversion mechanism is complex, which involves many signalling pathways in cancer progression.

### 3.1. Regenerative Capacity, Chemotherapy, and the Rise of CSCs

In treating cancer, a stem-cell transplant is used as part of the treatment, and often a stem-cell transplant involves treatment with a high dose of chemotherapy and radiation. After that, stem cells will be infused into the patient’s blood flow, and to suppress immunogenic reactions of the patient’s body they need to be treated with medication to suppress the immune system. This can lead to an increased risk of secondary cancer from a stem-cell transplant due to chemotherapy and radiation that may give rise to deoxyribonucleic acid (DNA) mutation leading to the formation of CSCs. Apart from DNA mutation, chemotherapy and radiation are able to damage normal tissue, which would require stem-cell migration and division at the damage site for repair, probably at a higher rate, creating a bigger risk for cancer. With the life-long regenerative capacity of these mutated cells following propagation, the number of stem-cell divisions has been reported to dictate the organ cancer risks [17]. Taken together, DNA mutation, stem-cell function, and tissue damage can very much increase the risk of cancer [18], which may be the major contributor to CSCs formation. Zhu et al. (2016) also reported that adult stem cells showed a higher chance of malignant transformation compared to stem cells from newborn animals [19]. This may indicate that the regenerative capacity of stem cells in adult humans may pose an increased risk of tumorigenicity, which may also explain the increase of cancer incidence in adulthood. This aspect needs to be further addressed whereby CSCs’ development may pose a biosafety issue in stem-cell therapy.

### 3.2. Homing Process

Apart from their regenerative capacity, stem cells are able to migrate, or home and regenerate in accordance to the niche, into other progenies of cells and maintain the stem-cell pool in the body [20]. The whereabouts of stem cells can be found in almost all organs and tissues. Most of these locations are at the peripheral blood, adipose tissue, umbilical cord blood, foetal tissues, lymph nodes, spleen, and thymus. From these sites, stem cells migrate through the circulatory system and travel to the targeted point regulated by chemokines and chemokines receptors [20,21]. Upon reaching the targeted sites, stem cells differentiate into cells committed to specific lineages depending on the microenvironment [22]. A similar homing process can be acquired by cancer cells, which leads to inflammatory reactions and enhances their metastatic ability. The initiation of metastasis, recurrence, and resistance of cancer cells towards treatment is due to CSCs.

Despite being small in population size, CSCs have a similar function as stem cells in terms of their ability for migration, self-renewal, and plasticity that contribute to heterogeneity and progression of cancer cells [23]. In the context of cancer, cells’ heterogeneity can be described as having phenotypic and functional variations of tumour cells that form within a tumour and different organs. Abilities to self-renew and differentiate into heterogeneous cell lineages of cancer cells in tumours have been accepted as the definition to describe CSCs [23]. These variations are characterised by specific markers including miRNA, which can be used for diagnostic and identification purposes, especially in the case of cell homing [24].

### 3.3. Cell Surface Markers Indicating CSCs

The stem-cell-like population known as CSCs has been previously characterised and identified by means of cell surface markers in tumours such as breast, colon, brain, pancreas, prostate, and hepatocellular carcinoma [9]. The discovery of CSCs has been organ-specific; hence, CSCs present a different set of markers and characterisation. The presence of these markers may be able to indicate surface markers’ expression, and genetic and morphological changes, particularly in stem cells undergoing a conversion mechanism towards CSCs. Cell surface markers may be used to indicate stem cell biosafety concerns in stem cell therapy. Although there has been a lot of interest in this area of research, there are still no conclusive or established outcomes to indicate specific surface markers for identifying CSCs. Their expression may be determined by molecular subtypes and metabolic signatures regulated by the microenvironment. In this section, we discuss some selected cell surface markers and secretomes that are potential candidates for identifying CSCs and cancer progression in relation to stem cells.

#### 3.3.1. CD90

Despite the possibility of becoming potential biomarkers of detecting CSCs in cancer, CD90 has multiple roles that can be contradictory, especially when it comes to cancer treatment. The CD90 can be either a suppressor or a promoter of cancer. CD90 is a GPI (N-glycosylated glycophosphatidylinositol) anchored cell surface protein. It has been found on T cells, endothelial cells, neurons, mesenchymal stem cells, fibroblast, and hematopoietic stem cells [25]. The functional roles of CD90 are activating T-cells, regulating neurite outgrowth in neurons, regulating the apoptosis of thymocytes and mesangial cells, modulating fibrosis, and upholding cells activity in migration, adhesion, and extravasation [25]. Its role is said to be dependent on environmental cues, which makes CD90 either a cancer promoter or suppressor. Chen et al. (2016) showed that the interaction of CD90 with the β3 integrin signalling pathway stimulates its suppressor activity on ovarian cancer [26]. Nevertheless, the overexpression of CD90 has been shown in many cancers and is a promoter for cancer development. Lobba et al. (2018) reported the oncogenic nature of CD90 in breast cancer and its involvement in the transformation of normal cells to malignant cancer [27]. CD90 specificity in cancer detection and development has been described elsewhere [28]. On the other hand, it has been reported that co-expression of CD90 is associated with an increase in development and progression of cancer. This has been observed in pancreatic intraepithelial neoplasias (PanIN) whereby CD90 and CD24 co-expression may specifically enable early detection of PanIN before it developed into pancreatic ductal adenocarcinoma (PDAC) [29].

#### 3.3.2. CD38

CD38 is a member of the ribosyl cyclase family that is expressed on the surface of immune cells and non-hematopoietic cells. It has been shown to be involved in the resistance mechanism of cancer cells towards chemotherapy. The immunosuppressive role of CD38 served as the escape mechanism for tumour cells from PD-1/PD-l1 blockade whereby it inhibited CD8+ T-cell activity through the adenosine receptor signalling pathway [30]. Expression of CD38 was found in 15–23% of cancer incidents with a strong correlation to the inflammatory mechanism in the tumour microenvironment [31]. Normally, the malignancies are haematological, such as multiple myeloma, NK/T cell lymphoma, and T-cell acute lymphoblastic leukemia [31]. In lung cancer, CD38 expression promotes cell growth by fortifying the cells’ anchorage ability, invasion, and xenograft growth in nude mice [31]. However, the development of malignancy in lung cancer was reduced with the deletion of ADP-ribose-acceptor hydrolase (ARH)-1 from the CD38 gene [31]. These findings support that CD38 plays a critical role in cancer prognosis and development. Although there has been much interest in this area of research, there are still no conclusive or established outcomes to indicate specific surface markers for identifying CSCs. Their expression may be determined by molecular subtypes [32].

#### 3.3.3. CD44

CD44 is a cell surface molecule and co-receptor for growth factors and cytokines. Shreds of evidence have suggested its involvement as a CSC marker in regulating cancer stemness [33,34]. It is involved in integration and transduction of cellular microenvironment signals of membrane-associated cytoskeletal proteins to regulate cell behavior, including CSC signalling and functional activation [35]. Williams et al. (2013) also reported that CD44 is involved in signalling integration of normal stem cells, CSCs, and pre-metastatic niches [35]. Chekhun et al. (2015) reported that CSCs CD44+/CD24- expression was detected in about 25.4% in patients with breast cancer of different molecular subtypes. Molecular subtype may determine the specificity and significance of CSCs on predicting the development of tumours in patients [36,37].

#### 3.3.4. CD133

CD133 is considered one of the best characterised CSC markers that is involved in CSC tumour-initiating capacity that can be suppressed through the p53-mediated CD133 inhibitory mechanism [38]. It can be found in abundance in normal tissue stem cells and as a putative CSC population that is influenced by epigenetic regulation subject to microenvironment cues [39,40]. The role of CD133 in cell epigenetic regulation may contribute to the genomic instability leading to stem-cell transformation [41]. On the other hand, CSCs can acquire unique metabolic signatures that may determine cancer development. Their distinct subtypes may play a significant role in organ-specific metastasis, and this was shown in lung metastasis where ALDH+/CD133+ and MET-like phenotype was expressed with oxidative metabolism [42].

#### 3.3.5. Bone Morphogenetics Proteins (BMPs)

BMPs is a diverse group of growth factor proteins that are associated with bone formation. It has been reported to modulate both cancer progression and suppression [43]. Various tumour microenvironment factors have been found to have strong dynamic interaction with BMPs including miRNAs, which also contribute to drug resistance in cancer cells [43,44]. In the presence of MSCs, BMP signalling was reported to increase the population of CSCs in ovarian cancer [45]. Although there is not much work on BMP roles in CSC mechanisms, interactions of BMPs with their antagonists and receptors have been associated with aggressiveness of tumours and establishment of cancer-cell metastasis mechanisms [46]. Interestingly, the dual role of BMPs in both cancer development and suppression has been reported depending on the type of BMPs in both cancer development and suppression and also on the type of BMP proteins [47,48]. This warrants more investigations on BMPs in relation to CSC mechanisms due to their diverse class of molecules associated with much physiological and disease development.

### 3.4. MicroRNAs

MicroRNAs (miRNAs) are non-coding RNAs that consist of a length of 21–25 nucleotides, making them the smallest RNAs. Despite the size, small miRNAs are the main regulators in the deactivation and degradation of a human gene after transcription by controlling the 3′-untranslated regions (3′-UTR). miRNAs have been reported to have an impact on cancer progression by regulating the progression of cancer stem-cells’ (CSCs) development. Studies have found links between abnormal miRNA expression and the tumorigenic ability of CSCs [49]. In pancreatic cancer, the use of gemcitabine (GEM) as treatment has faced limitations due to the chemo-resistant ability development of cancer cells. It was found that the downregulation of miRNA known as miR-205 increases the cancer stem cells’ proliferation, leading to human pancreatic cancer cells being more aggressive and chemo-resistant. Meanwhile, overexpressing miR-205 increases the sensitivity of pancreatic cancer cells towards GEM treatment by decreasing ALDH+ cells’ proliferation and downregulated chemo-resistant markers. Hence, regulating miR-205 may be the key to effective cancer treatment. The combination of GEM and miR-205 regulation was able to significantly reduce the proliferation of CSCs and 3D spheroids, making it an effective treatment for pancreatic cancer [50].

Another miRNA is known as miR-135b, an oncogene sited on chromosome 1q32.1 and encoded at the noncoding sections of the LEMD1 gene, giving it the ability to regulate cancer-cell growth rate [51]. It has a role in various cancer developments such as lung-cancer progression. Through the LZTS1 and Hippo pathway, miR-135b expression dysregulated the tumour suppressor components that increased the migration and invasive ability of lung-cancer cells. However, the impact of miR-135b expression happens with the modulations of DNA demethylation and inflammation signalling of nuclear factor-kappaβ [52]. Other external factors such as unbalanced circadian rhythm can also lead to cancer development [53]. The study also found that disruptions of circadian rhythm due to genetic, behavioural, and metabolic changes initiate the cancer signalling loop consisting of YY1 that activates miR-135b. The looping pathway eventually created a chemo-resistance ability of cancer cells. These studies indicate the role of miR-135 as a part of CSCs’ underlying mechanism and development. Understanding the role of miR-135 and its mechanism could lead to an effective treatment for cancer because in the case of pancreatic cancer stem cells (PCSCs), their growth and self-renewing ability take place when miR-135b is being suppressed. This leads to the inactivation of the AKT/mTOR pathway and increases the JADE-1 expression [54]. Hence, miRNAs do have a role in CSCs and may serve as markers in identifying and understanding the CSC mechanism.

### 3.5. Secretomes

Mesenchymal stem cell (MSC)-secreted cytokines and chemokines were found to modulate CSCs and cancer cells. Secretion of IL-1α and IL-1β by carcinoma cells stimulated the secretion of Prostaglandin E2 (PGE2) by MSC, which resulted in the stemness characteristics of colon cancer cells [47]. Conditional media derived from MSC cultures were reported to have higher levels of IL-6 and IL-8, which are associated with the progression of cancer cells by promoting invasion and proliferation of colorectal cancer cells via AMPK/mTOR-mediated NF-κB activation [48]. Depending on the cancer-cell types, the type and pattern of chemokines released by MSCs may determine the progression and interaction of cancer cells. These secretomes include CXCL1, CXCL5, 6 and 7, IL4, IL8, IL10, IL17b, S100A4, and EGF [55], which were also found to be involved in MSC migration to the tumour site [56].

### 3.6. Metabolic Changes and Characteristics

Although cell surface markers are commonly used in cancer research and diagnosis, they are normally insufficient to determine cancer prognosis as these marker expressions can be affected by the microenvironment in which they reside [57]. This can also promote the transformation and formation of CSCs. Metabolic prognosis is a new emergent field in cancer treatment that may indicate the possibility of CSCs having their metabolic signature that can be used for identification and to differentiate between stem cells and CSCs [58]. There have been several publications that addressed metabolic syndrome as a factor in supporting the survival of CSCs [59,60].

In metabolic regulation, mitochondria have been observed to be the main player in most cells in the body. Recent publications have shown that mitochondrial activity was reported to play an important role in a cancerous environment [61,62,63]. Similar metabolic changes and regulation influence stem cells’ survival and growth, which may provide critical events that initiate the conversion to CSCs. As a result of changes in metabolism, it can also lead to the release of inflammatory factors, creating a cancer-inducing niche under stressful metabolic conditions leading to initiation of CSCs [9].

## 4. Do Characteristics and Functional Similarities Easily Transform Normal Stem Cells to CSCs?

The nature and number of mutational changes have long been thought of as the basis of cancer initiation and development. However, some mutational changes do not result in cancer and are insufficient to cause cancer. Tomlinson et al. (1996) reported that only a small number of cancers are associated with mutations [64]. On the other hand, Versteg (2014) and Mack et al. (2014) showed that some cancers have no mutations at all [65,66]. Instead, the aforementioned investigators indicated that the mechanism leading to cancer is due to the conversion of normal cells into CSCs. There are many proposed models and theories that explain normal stem cell transformation or involvement in the development of cancer cells and CSCs. Overall, studies have reported that the presence of CSCs has been shown to contribute to the stemness of cancer cells, which allows the cells to have a high capacity for self-renewal and proliferation ability [67]. Similar to stem cells, CSCs also have the plasticity trait, which is the ability for CSCs to change characters such as by undergoing the epithelial-to-mesenchymal transition (EMT) during the embryonic development where the epithelial phenotype changes to a mesenchymal fibroblastoid phenotype [68]. The plasticity ability allows stem cell and CSC de-differentiation by augmentation of signalling pathways to ensure self-renewability and multipotency, depending on the niche [69,70,71,72]. However, augmentation of cell signalling pathways in EMT increases the risk of stem-cell mutations giving rise to CSCs. The mutation by EMT augmentation happens at the genetic level such as the overexpression of miR200, an EMT’s epigenetic regulator causing tumour progression. This indicates that EMT is a part of the dynamic manner of biological plasticity that will determine the cell’s fate during transition [73].

On the other hand, the connection between stem cells, cancer stem cells, and cancer cells can be presented by the cancer cell propagation models whereby there are three concepts known as the CSC model, clonal evolution model, and interconversion model that describe the transformation of normal stem cells to CSCs by promoting heterogeneity to support a cancerous microenvironment [74]. The CSC model follows a hierarchical mechanism whereby the differentiation ability of minor subpopulations of stem cells within the tumour leads to hierarchy of cell types that made the tumour [75]. Following the transformation of normal stem cells to CSC, a small population of CSC equipped with self-renewing and multipotent activity acts as a powerhouse for these normal stem cells to grow in symmetrical and symmetrically navigate towards malignant cells [76]. Depending on their microenvironment and niche, CSCs are able to acquire metastatic ability, which enables them to migrate from the tumour site and proceed to establish themselves in a new niche that gives rise to cancer cells [77,78].

## 5. The Influence of the Microenvironment in Promoting the Rise of CSCs

Stem cells live in a stem cell’s niche that is a microenvironment that plays a key role in controlling the maintenance and self-renewal of stem cells by secreting various paracrine factors or by direct cell–cell interaction that interferes with self-renewal and differentiation pathways. Tumour-specific microenvironments comprise cellular and soluble factors such as cancer-associated fibroblasts (CAFs), mesenchymal stem cells and endothelial cells as well as immune cells such as the macrophages, T-cells and natural killer (NK) cells, cytokines and growth factors, and the extracellular matrix (ECM) [79]. These factors collectively establish a network that maintains the stemness of CSCs as well as promoting the formation of new CSCs.

Fibroblasts play a significant role in the tumour-promoting mechanism. CAFs are one of the cellular components of the tumour microenvironment that drive tumour progression by secreting soluble factors, interacting with other cells, or modulating the composition of the extracellular matrix [80]. CAFs also secrete exosomes and communicate with the neighbouring cancer cells to stimulate migration, invasion, and metastasis formation [81]. Numerous studies have reported the autocrine and paracrine effects of CAFs on cancer cells. In breast cancer cells, CAF-derived cytokines facilitated glucose uptake by increasing the expression of the cell membrane-bound Glut-1 transporter level [82]. In prostate cancer, CAFs enhanced the growth potential of CSCs by increasing spheroid formation and the cancer cell proliferation index through paracrine signals [83,84]. CAFs have been reported to regulate cancer stemness by inducing a de-differentiation program mediated by Nanog, through the release of paracrine factors and activation of insulin-like growth factor 1 receptor (IGF1R) signalling [85]. In the hypoxic and hypo-nutritional tumour microenvironment, CAFs expressed higher levels of CD44, which promoted cancer a stem-phenotype including CSCs’ resistance to therapies [84]. In addition, Vermeulen et al. showed CAFs increased CSCs’ stemness via the paracrine activation of Wnt signalling pathways in colon cancer [85]. In hepatocellular carcinoma, CAF regulated liver CSCs through paracrine secretion of hepatocyte growth factor via activation of FRA1 in an Erk1,2-dependent manner [86] and the presence of TGF-β1, CAFs that enhanced cancer growth and metastasis [87].

Among the cells that play a role in the tumour microenvironment, mesenchymal stem cells (MSCs) have been demonstrated to crosstalk with the CSCs to promote tumour progression. MSCs can differentiate into CAF via a TGFβ1/Smad3-dependent mechanism [88] and promote metastasis of breast carcinoma cells [89,90]. In ovarian cancer, carcinoma-associated MSCs (CA-MSCs) promoted tumour growth by increasing the number of CSCs via BMP2 and BMP4 [46]. In addition, MSC-deregulated microRNAs caused downregulation of FOXP2 expression in breast-cancer cells and promoted CSCs’ propagation, tumour initiation, and metastasis [56]. Li et al. (2012) demonstrated that MSCs induced prostaglandin E2 (PGE2) secretion when they interacted with human colorectal carcinoma cells, and together with the cytokine network they act in a paracrine fashion on the carcinoma cells to induce activation of β-catenin signalling and formation of cancer stem cells [47].

The crosstalk between CSCs and immune cells in the tumour microenvironment is important to sustain stemness and promote the survival and plasticity of CSCs [77]. CSCs were reported to be involved with immune destruction and facilitate the establishment of an immunosuppressive tumour microenvironment through complex interactions with a broad range of immune cells, including NK cells, tumour-associated macrophages (TAMs), myeloid-derived suppressor cells (MDSCs), regulatory T cells (T-regs), cytotoxic T-lymphocytes (CTLs) and T helper (Th) cells [91]. In the innate immunity, CSCs were reported to be involved in the recruitment of macrophages into the tumour microenvironment [92] and to promote the macrophage polarization toward the M2 phenotype via secretion of colony-stimulating factors (CSFs), transforming growth factor β (TGF-β), macrophage inhibitory cytokine 1 (MIC-1) [93,94,95], CCL2 and CSF-1 [96]. The involvement of tumour-associated dendritic cells (TADCs) and their soluble factor CXCL1 on colon cancer cells was investigated [97]. TADCs promoted CSC properties, cell migration, and EMT by producing CXCL1 in a paracrine fashion. Some studies reported the ability of glioblastoma CSCs [98] and breast cancer CSCs [99] to escape tumour-infiltrating NK cells due to poor expression of MHC class I and NK ligands, which leads to tumour progression. Another mechanism through which cancer cells may evade the immune response by NK cells is the induction of apoptosis in microenvironmental immune cells through the interaction of CD95 (Apo1/Fas) with its ligands (CD95L). Ceppi et al. (2014) showed that stimulation of CD95R/L on cancer cells increases the number of CSCs and regulates their plasticity, thus reducing sensitivity to CD95-mediated apoptosis [100].

Recruitment of T lymphocytes across the tumour area is generally correlated with a successful clinical outcome. However, numerous studies have shown that infiltrating T cells can function in an immunosuppressive manner that promotes the progression of tumours. One of the main immune-suppressors are T-regs. T-regs that abundantly reside in the tumour microenvironment have been reported to be associated with poor prognosis of various types of cancers, including gastric, oesophageal, pancreatic, liver, and breast [101]. In a hypoxia environment, T-regs have been reported to induce the expression of IL-17, which promoted the expansion of CSCs through the activation of Akt and MAPK signalling pathways in colorectal cancer [102]. Xu et al. (2017) reported an interaction between T-regs and breast cancer cells that enhanced stemness of the cells by promoting the expression of Sox2, Nanog, and/or Oct4 markers, which resulted in an increase in sphere-forming capability [99]. A recent study reported that the CSCs were present in higher frequencies in patients with lymph node metastasis and were associated with the amount of tumour-infiltrating T-regs [103].

The influence of cytokines and growth factors in promoting the growth of CSCs has been reported extensively. CTLs-secreted IFN was reported to induce the proliferation and differentiation of leukaemia stem cells [104]. Meanwhile, IFN up-regulated the expression of the CSC markers CD24, CD44, and CD133 and promoted the migration and invasion of pancreatic ductal adenocarcinoma cells [105]. The role of IL-8 (CXCL8) on glioblastoma multiform CSCs has also been reported by Infanger et al. (2013) through a three-dimensional system of endothelial cells and CSCs that showed enhanced secretion of IL-8 by the endothelial cells that promoted the CSCs’ migration, growth, and stemness properties [98]. Regulatory crosstalk between IL-6/STAT3 inflammatory signalling and CSCs of the colorectal tumour showed enhanced stemness and progression toward malignancy in colorectal CSCs [106]. In pancreatic cancer, IL-22 promoted pancreatic cancer stemness via IL22RA1/STAT3 signalling, establishing the mechanism of regulation of cancer stemness in the tumour microenvironment [102].

Extracellular matrix (ECM) is a major structural component of the tumour microenvironment, and increased ECM deposition is associated with cancer development and progression [107]. ECM is formed by a complex structure of proteins, glycoproteins, proteoglycans, and polysaccharides. The ECM provides binding sites to CSC receptors via its structural proteins in the tumour microenvironment. The ECM serves as a niche to CSCs in the tumour microenvironment, which provided both structural and biochemical support to regulate proliferation, self-renewal, and differentiation of CSCs [108]. A previous study reported CSCs binding to CD44 (hyaluronan receptor) promoted the expression of stemness factors NANOG and SOX2 in breast and ovarian CSCs [109], and provided anchorage and homing sites for CSCs in pre-metastatic niches, initiating metastatic colonization and organotropism of the cancer cells [110]. Another study reported the involvement of ECM proteins in CSC maintenance in the tumour microenvironment and demonstrated that the periostin-integrin β3 signalling axis provided an important role in the maintenance of breast CSCs [111].

## 6. Clinical and Therapeutic Implications in Stem-Cell Therapy: Impact on Safety and Efficacy

Based on our understanding of the relationship between normal stem cells and CSCs, the implication of CSCs in stem cell therapy must be addressed should there be a risk of patients developing cancer following stem-cell therapy. Notwithstanding the quality and manufacturing considerations for cell therapy, there should also be an in-depth understanding of the biological impacts and the risks on safety and efficacy as more cell-based products and therapies enter the market with technologies that are always evolving and increasing in complexity [112]. As previously discussed, reports on characteristics and the relationship between normal stem cells and CSCs have shown the relevance of this issue which needs to be addressed. The correct question to ask may be “how do we analyse and mitigate the risks if there is potential for tumorigenecity?”.

A conventional approach to tumorigenicity testing such as a soft agar colony formation assay for cell-therapy products has been discussed thoroughly by Sato et al. (2019) [113]. The author points out that variation of testing outcomes is still an issue due to different methodologies used, whereby understanding their relevance, refinement and limitations needs to be further discussed. Hence, a harmonized procedure for regulations and technological aspects must be achieved in assessing the tumorigenicity of cell-therapy products. Regulatory bodies such as the European Medicines Agency (EMA) and the U.S. Food and Drug Administration (FDA) have come up with guidelines that provide a framework for the quality, safety, and efficacy evaluation that are considered for clinical-trial approval and marketing authorization, but specific tests for tumorigenicity risk analysis are yet to be provided [114]. In this regard, there is an imperative need for current tumorigenic assessment to be improved for successful clinical translation of cell-therapy products. Furthermore, certain issues need to be considered before clinical application and in developing new cell-based products or therapies. The aforementioned CSC-related markers may serve as a starting point in developing tumorigenic test assays in vitro in mitigating the risks of tumorigenicity, but they may not be specific and accurate enough in determining the significance of the outcome. The sensitivity of such assays is important to identify not just abnormalities, but also variations associated with technical difficulties and handling. In this regard, the development of a standardised and harmonised protocol for reference is important in safety and efficacy assessment. To develop such a protocol, science-based evidence and data generation are required to make an informed decision and to increase confidence in safety assessment for cell-based products and therapy. This initiative could also lead to the development of advanced technologies that can be used to understand and obtain appropriate data.

Emerging technologies such as the use of machine learning have been proposed as one of the approaches to assess the safety and efficacy of cell therapy [115]. However, it would require an advanced database to generate accurate prediction models for the assessment. Machine learning has been found to give meaningful information in developing strategies to optimize the efficacy of mesenchymal stem-cell treatment for tissue repair [116]. The machine learning use in this study adopted a neural network model to enable more accurate predictions of mesenchymal stem cell therapy treatment outcomes. Hence, the identification of key factors such as the number of cells to be implanted and the depth of the affected area was optimized for an effective cartilage repair and healing. Instead, Zhu et al. (2021) proposed the use of a neural network model with bright-field images in predicting the fate of neural stem cells whereby the identification method was able to generate data based on cell images obtained after 1 day of co-culturing [117]. The neural network model has been applied in pluripotent stem cells to distinguish between differentiating and undifferentiating cells with more than 99% accuracy [117]. The application of machine learning in predicting the stem cells’ fate and the outcome has been increasing with the potential to produce high sensitivity and accuracy in the data. This technique may serve as a tool in the assessment of stem-cell therapy safety and efficacy, particularly the risks of developing tumorigenicity.

## 7. Conclusions—Maneuvering the “Jekyll and Hyde” Situation

As evidence of the potential for stem-cell therapy grows, so do the related adverse effects in patients. This has been very much in debate due to unestablished standards and protocols to ensure safety and efficacy in stem-cell therapy. Further, there is still limitation as to what the stem cells can do, coupled with the fact that stem cell medicine is under-regulated, leading to violation of laboratory and manufacturing standards by the so-called “stem-cell clinics”. Unapproved treatments are sometimes advertised alongside promising ones with questionable protocols that violate basic safety principles. This has raised concerns, particularly with the ability of stem cells to promote cancer-like characteristics and progression in accordance to their niche microenvironment, which may be uncontrollable once it applied in vivo. In this regard, an online live-cell monitoring method was proposed to monitor cells for genetic modification and to process irregularities during the expansion phase [115]. Apart from that, there is also a need to ensure stem-cell potency—reliable metrics in the form of standardised potency assays utilising the biomarkers proposed by the International Society for Cellular Therapy (ISCT) to evaluate the therapeutic potential for a range of mesenchymal stem-cell products [114]. These are some of the methods that have been proposed in evaluating the safety and efficacy of stem-cell therapy. Whichever methods are used must be within or in parallel with regulatory control, and these regulations must be complied with by companies and clinics. On the other hand, monitoring methods, best practices, and an increase of regulatory enforcement would not just affect stem cell research, but most importantly the targeted patients, particularly those with debilitating diseases that could benefit from stem cell therapy. Implications could include medical technology advancement, wider healthcare options for patients, and better control of healthcare expenditure, but these methods could also simultaneously limit patients’ access to stem cell therapy as a result of increased regulatory authority. Monitoring of stem cell therapy, even in post-treatment, could ensure that the benefits continue to outweigh the risks for people who receive it. This may contribute to regulatory reform taking into account patients’ welfare, giving them access to proven safe and effective stem cell therapy.

Although stem cell therapy offers countless medical possibilities for various chronic diseases that led to a boom in the stem cell therapy market, there is a fine line between the pros and cons of the industry. This requires concerted efforts of all relevant parties, including healthcare professionals, scientists, industry players, regulators, and healthcare policymakers to manoeuvre, manage and regulate stem-cell therapy in ensuring safe, efficacious, ethical treatment and protect patients’ well-being and rights.

## Figures and Tables

**Table 1:** Common characteristics of normal and cancer stem cells (Adapted from Bapat et al., 2010).

Characteristics
Asymmetric division (self-renewal) that produces quiescent stem cells and a dedicated progenitor cell
A self-renewability by a similar signalling pathway (such as Wnt, Notch, MAPK, and sonic Hedgehog) and by BMI-1 at the epigenetic level
High telomerase activity that prolongs the cellular life span
Ability to form a hierarchy of cellular derivatives that includes progenitors and differentiated cells
Expression of similar surface receptors either as stem cell markers or associated with homing and metastases (such as CD133, c-kit, CXCR4, LIF-R, c-met, a6 integrin)
Preference for growth factor independence through secretion of growth factors and cytokines
Stimulation of angiogenesis through secretion of angiopoietin factors

**Table 2:** Normal SCs properties vs CSCs properties (Adapted from Cetin and Topcul 2012).

Properties	Normal SCs	CSCs
Self-renewal capacity	Extensive but limited	Extensive and indefinite
	Highly regulated	Highly dysregulated
Tissue or organ forming capacity	Organogenic	Tumorigenic
Cell differentiation capacity	Highly regulated	Highly dysregulated
The presence of cells	Rare in normal adult tissues	Infrequent or rare within tumours
Karyotyping	Normal	Abnormal
Replication state	Quiescent most of the time	Less mitotically active than other cancer cells
Identification	Can be easily identified based on established surface markers	Similar types of surface markers as the normal SC in the same tissue
Progeny capacity	Normal with limited proliferative potential	Phenotypic variation

## Data Availability

Not applicable.
